# The War and Nervous Diseases

**Published:** 1914-11-28

**Authors:** Edwin L. Ash

**Affiliations:** 24 Harley Street, W.


### THE EDITOR'S LETTER-BOX.

### The War and Nervous Diseases.

*To the Editor of* The Hospital.

Sir,—Referring to the remarks on this subject in your issue of November 21, it is important that the promoters of any scheme for the care of persons suffering from nervous shock, consequent upon active military service, should not handicap themselves by any misconception as to the sort of provision they must make. As indicated by his appeal, Lord Knutsford appears to consider that the existing hospitals for nervous diseases in London cannot under any circumstances provide accommodation that will give proper rest and isolation to the unfortunate individuals whose interests he has at heart. In his reply to the criticisms of Sir Frederick Macmillan, chairman of the National Hospital for the Paralysed and Epileptic, Lord Knutsford writes (*Times*, November 13, 1914) that, although this institution could in a week fit up a ward with curtained cubicles, "this is not at all what is wanted, and would be actually harmful to some patients." One would imagine that after the din and turmoil of the modern battlefield the nerve-wracked soldier would find even a bed in an ordinary London hospital ward a welcome haven of rest. How much more so, then, would he feel the benefit of rest in a separate cubicle, particularly were he placed in a ward divided into separate compartments, and in charge of competent nurses who would see to it that the requisite restful conditions prevailed throughout. It is not to be anticipated that patients of the kind under consideration will be of the "noisy" type. Clearly some separate provision will in any case have to be made for noisy and delirious invalids; for one knows that even when placed in a separate room a noisy patient can disturb a whole floor.

I am quite certain that in practice it will be found that a month or six weeks' rest, with massage and electricity—and possibly psychotherapy in certain selected cases—will suffice so far to restore strength and self-confidence that the neurasthenic victims of the present Continental struggle will then be able to be sent into the country, there to undergo the long and tedious convalescence inseparable from this class of illness. And it is very probable that, as suggested in your aforementioned leader, the metropolitan hospitals for nervous diseases will be able to accommodate for treatment in the early stages a very fair proportion of men requiring it. So far so good. But, Sir, you conclude by saying that "we feel there is much to be said, however, in favour of the provision of a quiet home in the country," whilst the fact remains that not one home, but many, will probably be required. I very much doubt whether any scheme based on the provision of one large home for convalescent nervous cases will in the end touch more than the fringe of the matter, as the number of sufferers requiring rest and nerve treatment in the country will probably be very large, whilst in each individual instance the time of stay will be lengthy.

I would submit, then, that instead of collecting funds for equipping one or even several large country houses for the accommodation of a number of cases in each, it will be better to arrange for the placing of them in the hands of those who will be kind enough to offer one or two rooms in their houses for this purpose. From letters I have already received, I feel sure that it would not be difficult to obtain the offer of a great many rooms in private houses where for nothing, or at a nominal cost to cover bare expenditure, our patients could be accommodated in charge of an expert nurse. Under the circumstances the chief demand on any fund raised would be the payment of nurses—male and female—having special experience of neurasthenia and the neuroses generally. As a matter of fact, I think that a number of nurses would be found to volunteer their services, or at any rate to offer them at greatly reduced rates, if required. The medical care of these convalescents would devolve upon local medical men, who would, I am sure, be ready to supervise the treatment without charge.

My interest in the matter has already brought me offers of accommodation and professional service which would enable me to arrange, if necessary, for the care of a small number of neurasthenic soldiers, whilst I have every reason to believe that a definite appeal for help would bring in many more offers of a similar kind in regard to medical attendance, nursing, and housing. I should be glad to hear from fellow-workers also interested in this matter.—Believe me, faithfully yours,

Edwin L. Ash, M.D.

24 Harley Street, W.
November 23, 1914.

